# Association between gestational exposure and risk of orofacial clefts: a systematic review and meta-analysis

**DOI:** 10.1186/s12884-023-06104-4

**Published:** 2023-12-01

**Authors:** ZhiMeng Huang, JinZhun Wu, Yue Qiu, Jiayan Lin, Wanting Huang, Xiaohui Ma, Huifen Zhang, Xiaoqing Yang

**Affiliations:** 1 Department Pediatrics, Women and Children’s Hospital, School of Medicine, Xiamen University, Fujian Province, 361000 China; 2 Key Laboratory of Urban Environment and Health, Institute of Urban Environment, Chinese Academy of Sciences, University of Chinese Academy of Sciences, Fujian Province, 361000 China

**Keywords:** Air pollution, Cleft palate, Cleft lip, Gestational exposure, Meta-analysis

## Abstract

**Background:**

The occurrence of orofacial Clefts (OFCs) is a congenital disease caused by many factors. According to recent studies, air pollution has a strong correlation with the occurrence of OFCs. However, there are still some controversies about the current research results, and there is no relevant research to review the latest results in recent years.

**Objective:**

In this paper, the authors conducted a systematic review and meta-analysis to explore the correlation between ambient air pollution and the occurrence of neonatal OFCs deformity.

**Methods:**

We searched Pubmed, Web of science, and Embase databases from the establishment of the database to May 2023. We included observational studies on the relationship between prenatal exposure to fine particulate matter 2.5 (PM2.5), fine particulate matter 10 (PM10), sulfur dioxide (SO2), nitrogen dioxide (NO2), ozone (O3), carbon monoxide (CO) and the risk of cleft lip (CL), cleft palate (CP), cleft lip with or without palate (CL/P). the Newcastle-Ottawa quality assessment scale (NOS) was used to evaluate the quality of the literature. Funnel plot and Egger’s regression were used to verify the publication bias. Random effect model or fixed effect model was used to estimate the combined relative risk (RR) and 95% confidence interval (95%CI).

**Results:**

A total of eleven studies were included in this study, including four cohort studies and seven case-control studies, including 22,453 cases of OFCs. Ten studies had low risk of bias and only one study had high risk of bias. Three studies reported that PM_2.5_ was positively correlated with CL and CP, with a combined RR and 95%CI of 1.287(1.174,1.411) and 1.267 (1.105,1.454). Two studies reported a positive correlation between O_3_ and CL, with a combined RR and 95%CI of 1.132(1.047,1.225). Two studies reported a positive correlation between PM_10_ and CL, with a combined RR and 95%CI of 1.108 (1.017,1.206). No association was found between SO_2_, CO, NO_2_ exposure during pregnancy and the risk of OFCs.

**Conclusion:**

The results of this study showed that there was a significant statistical correlation between exposure to PM_10_, PM_2.5_, O_3_ and the risk of OFCs in the second month of pregnancy. Exposure assessment, research methods and mechanisms need to be further explored.

**Supplementary Information:**

The online version contains supplementary material available at 10.1186/s12884-023-06104-4.

## Introduction

Birth defects, also known as congenital malformations or congenital diseases, are due to external environmental factors, genetic factors or both leading to structural, functional and metabolic abnormalities in the process of growth and differentiation [1]. OFCs is a common type of birth defect disease. In the global survey report on the prevalence and disease burden of OFCs from 1990 to 2017, about 10.8 million children with OFCs were found, and most of them were distributed in low- and middle-income countries [2].

The incidence of OFCs is very different in countries around the world. From 2006 to 2018, a cross-sectional study of 5.74 million live births in South Korea found that the incidence of OFCs was 19.6/10,000 [3]. From 2007 to 2011, the incidence of CL alone in the United States was 3.1/10,000, the incidence of OFCs was 5.6/10,000, and the incidence of CP alone was 5.9 /10,000 [4]. Compared with other regions, China has a higher incidence of OFCs. From 2015 to 2018, the southern region of China investigated the occurrence of OFCs for four years. It was found that the incidence of OFCs was 7.55/10,000, and the incidence of male children was higher than that of female children. It is considered to be related to the serious pollution of China and the imbalance of economic and medical development [5]. However, the incidence of OFCs in South Africa from 2015 to 2016 was only 3.2/10,000, which may be related to the backward development of the public health system, resulting in a large number of cases not included in the study [6].

Children with OFCs will have many complications due to oral structure problems, such as difficulty in breastfeeding, increased incidence of anemia, and language development disorders [7, 8]. Compared with normal children, children with OFCs are more likely to die from cardiovascular disease [3]. In the Global Burden of Disease (GBI) survey, the disease burden of OFCs from 1990 to 2017 was 652,084 disability-adjusted life years (DALYs). Most of the burden of disease costs occurs in low-and middle-income countries, accounting for about 94.1% of the total cost. This indicates that the occurrence of OFCs will increase the burden of medical resources and social economy [2]. From 2013 to 2018, a survey conducted in South Korea on children with OFCs surgery showed that although the length of hospital stay was slowly decreasing, the number of operations remained basically unchanged, and the cost of hospitalization gradually increased [9]. Relevant studies have shown that air pollution increases the risk of birth defects. In 2005, Beate Ritz et al. used a case-control study to find that with the increase of O_3_ exposure dose, the risk of aortic valve defect, pulmonary valve malformation and conotruncal defect increased in a dose-response relationship [10].

A retrospective control study in Ohio, USA, showed that for every 10 µg/m^3^ increase in PM_2.5_ concentration in the first month of pregnancy, the OR and 95%CI of birth defects were 1.09 (1.01,1.18) [11]. Studies have also shown that air pollutants can increase the risk of diseases such as premature birth and low birth weight [12, 13]. In order to analyze the relationship between air pollutants and the occurrence of OFCs, the author collected the research literature of common air pollutants such as PM_2.5_, PM_10_, CO and OFCs, and conducted systematic review and meta-analysis to explore the possible correlation between common environmental air pollution and the occurrence of neonatal OFCs.

## Materials and methods

### Search strategy

According to the Preferred Reporting Items for Systematic review and Meta-analysis (PRISMA) [14], PubMed, Embase and Web of Science databases were searched with the keywords of “air pollution”, “particulate matter”, “sulfur dioxide”, “nitrogen dioxide”, “carbon monoxide”, “ozone”, “cleft lip”, “orofacial Clefts”, “cleft palate”, “cleft lip with or without cleft palate”. See supplementary materials for retrieval criteria. The search time was from the establishment of the database to May 30,2023.

### Study selection

Studies were selected according to the following criteria: [1] The study included exposure to PM_10_, PM_2.5_, SO_2_ and other common air pollutants during pregnancy; [2] The types of included studies were cohort studies and case-control studies, while reviews, case reports, and conference proceedings were not included [3]. The study data included OR, RR and 95%CI [4]. All the literature is in English. Two authors independently searched the literature and resolved the differences through discussion.

### Data extraction and quality assessment

Two authors extracted the following information from the retrieved literature: first author, study design method, publication year, study location, study duration, sample size, exposure time, exposure method, related covariates, OR, RR and 95%CI. The Newcastle-Ottawa quality assessment scale (NOS) was used by two authors to independently evaluate the quality of the literature, with a total score of 9 points. There were three main evaluation criteria: selection of subjects, comparability between groups, and measurement of exposure factors [15]. If at least two of the three evaluations meet the evaluation criteria, the study will be considered to have a low risk of bias [16, 17].

### Statistical analysis

First, we used the funnel plot and Egger’s test to verify the publication bias, and ***P*** < 0.1 indicated that there was publication bias. Secondly, when extracting the RR value, we chose the RR value in the single pollutant model, because not all studies have adjusted other pollutants as covariates, and there is a certain collinearity between multiple pollutants [18]. Heterogeneity was assessed by chi-square test and inconsistency coefficient ***I***^***2***^. When ***P*** > 0.1 and ***I***^***2***^ ≤ 40, the fixed effect model was used to evaluate the RR value. Otherwise, we use the random effects model [19]. Finally, we selected the literature with low quality in the group with high heterogeneity (***I***^***2***^ > 40) and no publication bias for sensitivity analysis.

In addition, the literature we included has the problem of different exposure time. Excluding the potential impact of exposure time, we chose the most critical period of facial partialization and development. The 5–10 weeks of gestation and the second month of gestation mentioned in the most literature are the most critical period for the differentiation and development of OFCs. We use these periods as an exposure period [20, 21]. All analyses were performed using STATA 16.0. Bilateral test was used, and ***P*** < 0.05 indicated that the difference was statistically significant.

## Results

### Literature selection

**Fig. 1:**
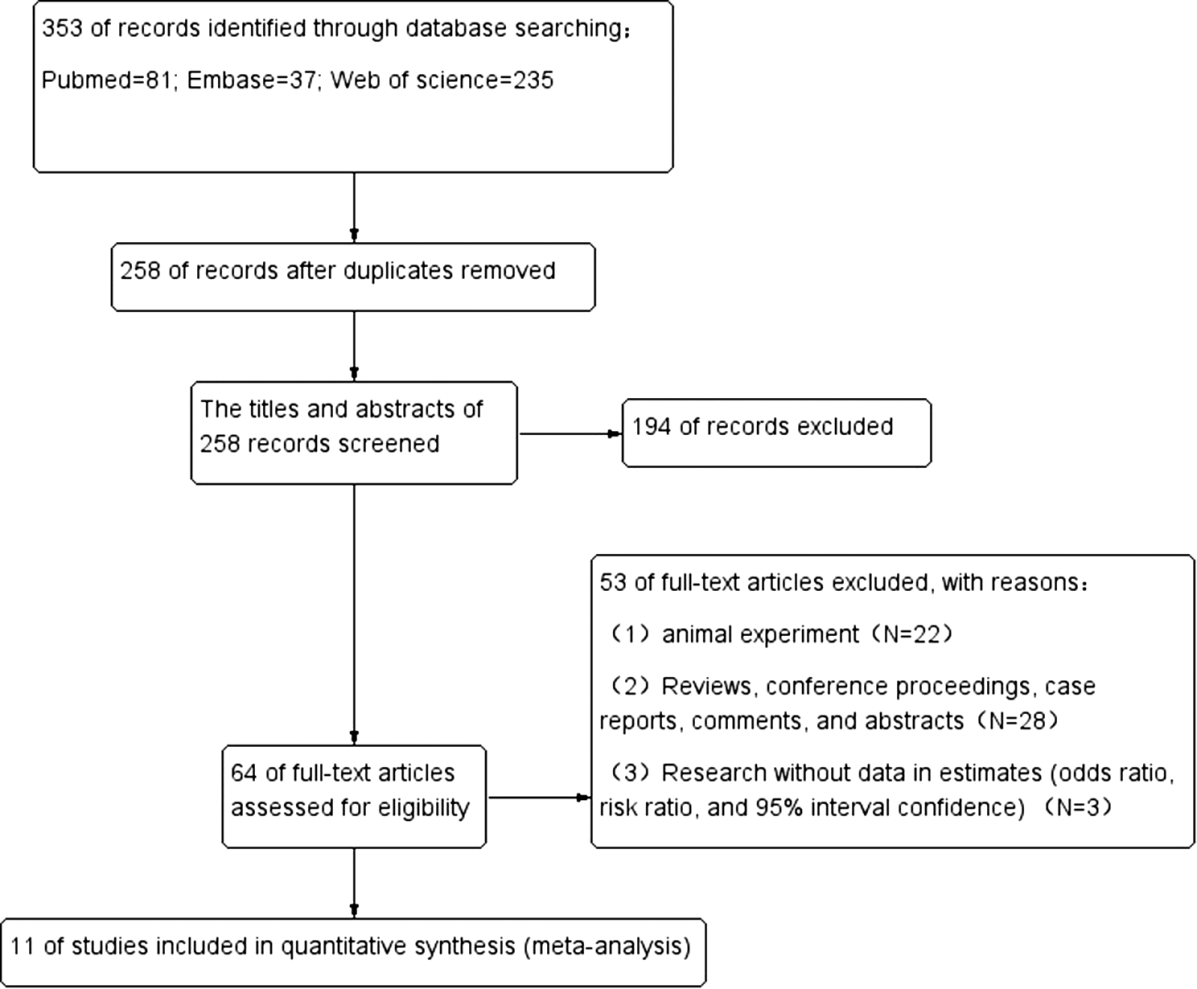
Flow chart of the study selection process

By searching the database, we retrieved 353 articles, deleted 95 duplicate articles, read the titles and abstracts of the remaining 258 articles, and deleted 194 articles according to the criteria set above. The remaining 64 articles were further evaluated. After full-text reading, 53 articles that did not meet the requirements were excluded, including 22 articles on animal experiments, 28 articles on reviews, conferences, and case reports, and 3 articles on lack of data evaluation. Finally, 11 articles were included in the study [10, 22–31](Fig. 1).

### Study characteristics

In this paper, 11 articles were included, including a total of 22,453 children. All research data were from the birth defect registration system and hospital monitoring system, including all children with live births, stillbirths or selective termination of pregnancy. Of the 11 studies, 7 were case-control designs and 4 were cohort studies. 5 studies were from the United States, 4 from China, and 2 from other regions and countries. The study time ranged from 1997 to 2018. The time window of air pollutant exposure was mainly concentrated in the first 3 months of pregnancy, and the risk of OFCs was mainly concentrated in the first 2 months of pregnancy (Table 1).

**Table 1:** Characteristics of the studies in the review

author(year)	Exposure period	Pollutant(s)	Exposure assessment	Study design	Statistical method	Adjustment variables	Data source	Period	Total Number	Case number	Location
Gilboa SM et al.(2005)	Weeks 3–8 of pregnancy	PM10、O3、CO、SO2、NO2	Air pollutants data from the nearest monitoring station	Case-control	Logistic regression models	Alcohol consumption or smoking during pregnancy, gravidity, maternal age, maternal education, maternal illness, maternal race/ethnicity, parity, plurality, prenatal care, season of conception and so on.	The Texas Birth Defects Registry provided data on birth defect diagnoses for 7,381 livebirths and fetal deaths of infants	1997–2000	607500	293	USA
Hwang BF et al.(2008)	First trimester	PM10、O3、CO、SO2、NOx	The inverse distance weighting method	Case-control	Logistic regression models	Sex of infant, maternal age, plurality, gestational age and season of conception.	The Taiwanese Birth Registry from 2001 through 2003	2001–2003	721289	653	Taiwan
Hansen CA et al.(2009)	Weeks 3–8 of pregnancy	PM10、O3、CO、SO2、NO2	Air pollutants data from the nearest monitoring station	Case-control	Conditional logistic regression	neonate gender	The Queensland Health Perinatal Data Collection Unit	1997–2004	150308	none	Australia
Marshall EG et al.(2010)	Weeks 3–8 of pregnancy	PM2.5、PM10、O3、CO、SO2、NO2	Air pollutants data from the nearest monitoring station	Case-control	Logistic regression models	mother’s age, race, ethnicity, smoking and drinking alcohol during pregnancy, and season of conception	The New Jersey Department of Health and Senior Services (NJDHSS) Special Child Health Services registry.	1998–2003	690000	717	USA
Zhu Y et al.(2015)	Weeks 3–8 of pregnancy and the 3 months pre-pregnancy	PM2.5、PM10、O3、CO、SO2、Nox	The inverse distance weighting method	Cohort	Logistic regression models	site/region, maternal age, race, marital status, insurance, pregnancy body mass index, nulliparity, smoking and/or alcohol consumption during pregnancy and so on.	The Consortium on Safe Labor included 12 clinical centers	2002–2008	188102	159	USA
Tanner JP et al. (2015)	Weeks 3–8 of pregnancy	PM2.5、benzene	The inverse distance weighting method	Cohort	Multivariable Poisson regression	Maternal race/ethnicity, Maternal age, Maternal education, Maternal marital status, Parity	The Florida Birth Defects Registry	2000–2009	2123874	923	USA
Zhou Y et al.(2017)	Weeks 5–10 of pregnancy	PM2.5、O3	The Bayesian downscaler model	Case-control	Conditional logistic regression	infant sex, maternal race and ethnicity, maternal education, smoking status during pregnancy, mother’s age, and parity.	The National Birth Defects Prevention Network (NBDPN) and the Centers for Disease Control and Prevention (CDC).	2001–2007	4697523	7035	USA
Zhao J et al.(2018)	First trimester	PM2.5、PM10、O3、CO、SO2	Air pollutants data from the nearest monitoring station	Cohort	Multivariate logistic regression models	maternal ages, education levels, infant sexes, parities, the season of conception, the air temperature, the humidity, and the air pressure.	The Wuhan Maternal and Child Health Management Information System	2011–2013	108167	133	China
Wang L et al.(2019)	First trimester	SO2、NO2、PM10	The ordinary block kriging	Cohort	Poisson generalized additive model	long-time trend, seasonality, temperature and relative humidity	The Xi’an Birth Defects Monitoring System	2010–2015	755551	8865	China
Jiang W et al. (2021)	First trimester	CO、NO2、SO2、O3、PM2.5、PM10	Air pollutants data from the nearest monitoring station	Case-control	Multivariate logistic regression models	maternal age、maternal educational level、gravidity, infant sex, plurality, temperature and relative humidity.	The hospital based birth defect monitoring (HBBDM) system of Hunan Province and the electronic medical records (EMR)	2015–2018	none	589	China
Liu FH et al.(2021)	First trimester and the 3 months pre-pregnancy	SO2	The mean concentration of all air monitoring stations in the city	Case-control	Univariate logistic regression	maternal age, education, season of conception and the mean levels of PM10, as well as NO2 during the same period.	14 city institutions of maternal and child health.	2010–2015	1365000	3086	China

### Quality assessment

The NOs was used to evaluate the quality of the included articles. The scores were between 6 and 8 points, and the average score of the NOs was 7 points. The specific scores are shown in Table 2. For PM10, groups with highest heterogeneity was “PM10-CP”, ***I***^***2***^ was 79.0%. For PM2.5, groups with highest heterogeneity was “PM2.5-CL/P”, ***I***^***2***^ were 86.9%. For O_3_, groups with highest heterogeneity was “O_3_-CP”, ***I***^***2***^ were 83.7%. No publication bias was found in the results of all groups. The heterogeneity test and publication bias of each group were detailed in Table 3.

**Table 2:** NOs score of studies included in the systematic review and meta-analysis

Study	Selection	Comparability	Outcome/Expose	Total score
Wang L et al.	3	2	2	7
Jiang W et al.	4	1	2	7
Zhao J et al.	4	2	2	8
Marshall EG et al.	4	1	2	7
Hansen CA et al.	4	1	2	7
Zhu Y et al.	3	1	2	6
Zhou Y et al.	4	2	2	8
Hwang BF et al.	4	1	2	7
Liu FH et al.	4	2	2	8
Gilboa SM et al.	3	2	2	7
Tanner JP et al.	4	1	2	7

**Table 3:** Summary of meta-analysis of studies on air pollutant exposures and OFCs

Air pollutants and OFCs	Studies Included	Summary RR and (95% CI)	I2 (%)	Egger’s test P-value
PM10				
CL	22、23、24、27	1.021、( 0.978, 1.066)	68.00%	0.252
CP	10、22、23、24、25、29	1.029、( 0.947, 1.117)	79.00%	0.446
CL/P	22、24、25、29	1.108、(1.017, 1.206)	44.30%	0.652
PM2.5				
CL	22、30	1.287、(1.174, 1.411)	0.00%	0.826
CP	22、23、25、26、30	1.267、(1.105, 1.454)	72.40%	0.704
CP(sensitivity analysis)	22、23、26、30	1.226、(1.088, 1.381)	63.50%	0.72
CL/P	22、23、25、26、30	1.136、(0.979、1.317)	86.90%	0.362
SO2				
CL	22、23、24、27	1.070、(0.840, 1.364)	83.70%	0.424
CP	21、22、23、24、25、29	0.887、(0.743, 1.058)	60.30%	0.344
CL/P	21、22、24、25、28、29	1.111、(0.956, 1.291)	81.50%	0.205
O3				
CL	22、24、27	1.132、(1.047, 1.225)	67.90%	0.793
CP	21、22、23、24、25、26、29	1.026、(0.885, 1.190)	82.20%	0.638
CL/P	21、22、23、24、25、26、29	0.993、(0.961, 1.025)	51.60%	0.774
CO				
CL	22、24、27	1.057、(0.865, 1.291)	96.80%	0.824
CP	21、22、23、24、25、29	1.048、(0.714, 1.539)	93.10%	0.737
CL/P	21、22、23、24、25、29	1.175、(0.936, 1.475)	92.90%	0.461
NO2				
CL	24、27	0.976、(0.831, 1.147)	66.60%	0.884
CP	20、21、23、24、25、29	1.201、(0.828, 1.742)	93.20%	0.949
CL/P	21、23、24、25、29	1.187、(0.938, 1.503)	85.30%	0.16

### Data synthesis

#### Overview of meta-analyses

For PM_10_, PM_2.5_, SO_2_, O_3_ and other air pollutants, we studied the relationship between these pollutants and OFCs. In the preliminary analysis, PM_10_ was positively correlated with CL/P, with RR and 95%CI of 1.108(1.017,1.206). PM_2.5_ was positively correlated with CL and CP, with RR and 95%CI were 1.287(1.174,1.411) and 1.267 (1.105,1.454). O_3_ was positively correlated with CL, RR and 95%CI were 1.132(1.047,1.225). There was no significant correlation between residual air pollutants and CL, CP, CL/P (Table 3).

#### Association between PM10 and OFCs

Seven studies reported the association between PM_10_ exposure during pregnancy and the risk of OFCs. Four of the seven studies reported the association between PM_10_ exposure during pregnancy and the risk of CL. Six of the seven studies reported the association between PM_10_ exposure during pregnancy and the risk of CP. Four of the seven studies reported the association between PM_10_ exposure during pregnancy and the risk of CL/P.

Among the four studies reporting the association between PM_10_ and the risk of CL/P, Zhao J et al. observed a significant positive correlation between PM_10_ exposure during pregnancy and the risk of CL/P (RR = 1.11, 95%CI= (1.00,1.23)) [24]. The pooled RR = 1.108, 95%CI= (1.017,1.206), heterogeneity was low (***I***^***2***^ = 44.3%), (Table 3), Egger’s test did not detect publication bias (***P*** = 0.652). In the study of the association between PM_10_ and the risk of CL/P, no significant association between PM_10_ and the risk of CL/P was found.

#### Association between PM2.5 and OFCs

Five studies reported the correlation between PM_2.5_ exposure during pregnancy and the risk of OFCs. Two of the five articles studied the relationship between PM_2.5_ exposure during pregnancy and the incidence of CL. Five studies studied the relationship between PM_2.5_ exposure during pregnancy and CP, CL/P.

In the literature on the association between PM_2.5_ exposure during pregnancy and the risk of CL, Zhao J et al. observed a significant positive correlation between PM_2.5_ exposure during pregnancy and the risk of CL, with RR = 1.29, 95%CI= (1.171,1.421), and the combined RR = 1.287, 95%CI= (1.174,1.411) [24]. Similarly, in the literature on the association between exposure to PM_2.5_ during pregnancy and the risk of CP, Zhao et al., Zhu et al., Zhou et al. observed a significant positive correlation between PM_2.5_ and the risk of CP [24, 27, 28]. The combined results of 5 studies showed that for every 1 µg/m^3^ increase in PM_2.5_ exposure in the second month of pregnancy, the risk increased by 26.7%, 95%CI= (1.105,1.454) and the heterogeneity was high(***I***^***2***^ = 72.4%), Egger ‘s test did not detect publication bias (***P*** = 0.704) (Table 3). Due to the poor quality of Zhu Y et al. ‘s research literature, we excluded it. The final study combined RR = 1.226, 95%CI= (1.088,1.381), ***I***^***2***^ = 63.5%, Egger’s test (***P*** = 0.720), heterogeneity decreased, and no publication bias was detected. In the association study between PM_2.5_ and the risk of CL/P, no significant association was found between PM_2.5_ and the occurrence of CL/P.

#### Association between SO2 and OFCs

Eight studies reported the correlation between exposure to SO_2_ during pregnancy and the risk of OFCs. Four of the eight articles studied the relationship between exposure to SO_2_ during pregnancy and the incidence of CL. Six of the eight articles studied the relationship between SO_2_ and CP, CL/P.

In the literature on the association between SO_2_ exposure during pregnancy and the risk of CL/P, Jiang W et al. observed a significant positive correlation between SO_2_ exposure during pregnancy and the risk of CL/P, with RR = 1.350, 95%CI=(1.140,1.610), and the remaining correlation was found in the remaining five articles [23]. The combined RR = 1.111, 95%CI= (0.956,1.291) was not statistically significant. In the study of the association between SO_2_ and the risk of CL/P, no significant association was found between SO_2_ and the occurrence of CL/P (Table 3).

#### Association between O3 and OFCs

Eight studies reported the correlation between exposure to O_3_ during pregnancy and the risk of OFCs. Three of the eight articles studied the relationship between exposure to O_3_ during pregnancy and the incidence of CL. Seven of the eight articles studied the relationship with CP, CL/P.

In the literature on the association between exposure to O_3_ during pregnancy and the risk of CL, Zhao J et al. and Hwang BF et al. observed a significant positive correlation between exposure to O_3_ during pregnancy and the risk of CL, with RR of 1.120 and 1.220, 95%CI of (1.020,1.220) and (1.030,1.460), respectively. The combined RR of the three studies was 1.132, 95%CI= (1.047,1.225), and the heterogeneity was moderate(***I***^***2***^ = 67.9%), Egger ‘s test (***P*** = 0.793) did not detect publication bias (Table 3) [24, 29]. In the literature on the association between exposure to O_3_ during pregnancy and the risk of CP, two articles reported a significant positive association, five articles reported an insignificant association, and the combined effect estimate was close to one, but not statistically significant. In the study of the association between O_3_ and the risk of CL/P, no significant association between O_3_ and the risk of CL/P was found (Table 3).

#### Association between CO and OFCs

Seven studies reported the association between exposure to CO during pregnancy and the risk of OFCs, three of the seven articles studied the relationship between exposure to CO during pregnancy and the risk of CL, and six articles studied the relationship between exposure to CO during pregnancy and the risk of CP, CL/P.

In the literature on the association between CO exposure during pregnancy and the risk of CL, Zhao J et al. observed a significant positive correlation between CO exposure during pregnancy and the risk of CL, with RR = 1.240, 95%CI= (1.110,1.40) [24]. The combined RR = 1.057, 95%CI= (0.865,1.291) was not statistically significant. In the literature on the association between CO exposure during pregnancy and the risk of CP, Zhu Y et al. observed that there was a significant positive correlation between CO exposure during pregnancy and the risk of CP. For every 1 µg/m^3^ increase in CO exposure in the second month of pregnancy, the risk increased by 174%, 95%CI= (1.620,4.620), and no significant correlation was found in the remaining articles [27]. The combined RR = 1.048, 95%CI= (0.714,1.539) was not statistically significant. No significant association was found between CO and the risk of CL/P (Table 3).

#### Association between NO2 and OFCs

Seven studies reported the correlation between NO_2_ exposure during pregnancy and the risk of OFCs. Two of the seven articles studied the relationship between NO_2_ exposure during pregnancy and the incidence of CL. Six and five studies studied the relationship with CP, CL/P, respectively.

In the literature on the association between NO_2_ exposure during pregnancy and the risk of CP, three articles reported a significant positive association, and the other reported an insignificant association. The combined RR = 1.201, 95%CI= (0.828,1.742) was not statistically significant. Jiang W et al. observed a significant positive correlation between NO_2_ exposure during pregnancy and the risk of CL/P, with RR = 1.48, 95%CI=(1.250,1.750) [23]. The combined effect estimates were close to one, not statistically significant. No significant association was found between NO_2_ and the risk of CL.

## Discussion

Our results showed that there was a significant positive correlation between exposure to PM_2.5_, PM_10_, O_3_ in the second month of pregnancy and the risk of CL/P. Among them, PM_10_ was associated with an increased risk of CL/P. This is consistent with the results of Rao A et al. ‘s previous meta-analysis that O_3_ increases the risk of OFCs [32].

In 2016, the results of the global death factor survey showed that air pollutants were the sixth leading cause of death, and 7.5% of global deaths were attributed to ambient air pollution. The countries with higher deaths included China and India [33]. PM can be emitted directly from sources such as construction sites, roads, fields, chimneys, or formed by complex reactions of chemicals such as sulfur dioxide and nitrogen oxides. PM consists of hundreds of different chemicals [34]. Relevant studies have found that particulate pollutants can freely pass through the placenta and accumulate on the side of the fetus by comparing the levels of particulate pollutants on both sides of the placenta under different exposure levels of particulate pollutants [35]. PM may exert its adverse effects by directly acting as a pro-oxidant or free radical generator for lipids and proteins, promoting oxidative stress and inducing inflammatory responses [36, 37].

Ozone (O_3_) is a photochemical environmental pollutant affected by climate. It is easy to form when the temperature fluctuates greatly [38]. Studies have shown that high concentrations of ozone pollution can lead to adverse health effects and increase the morbidity and mortality of respiratory and cardiovascular systems [39–42]. This is consistent with our results that there is a significant positive correlation between exposure to PM_2.5_, PM_10_, O_3_ and the risk of OFCs during the critical period of pregnancy.

There are many reasons for the occurrence of OFCs, including genetics, environment and their combined effects. The combination of genetics and environment is called “epigenetics” [43]. Related studies have shown that low-income families, pesticide exposure history, smoking during pregnancy, gestational diabetes, and heavy metal exposure history such as Pb(lead), Cd (cadmium), and Sr(strontium) are all high-risk risks of OFCs. The reasonable supplement of vitamin, potassium and calcium during pregnancy is a protective factor to reduce the occurrence of OFCs [44–47]. However, due to the limitation of the number of studies included in this paper and the difference in the quality of the literature, Hansen CA et al. only adjusted the gestational age as a covariate, and lacked corrections for other covariates, which would lead to deviations in the analysis results [26].

Whether the address change during pregnancy will affect the accuracy of the results, many scientists have studied it. Two cohort studies in the United States have shown that the address registered at birth can well replace the residential address during pregnancy. The exposure concentration of air pollutants will not be significantly different due to the difference between the registration address at birth and the residence address during pregnancy [48, 49]. But a cohort study in China ‘s Gansu province found that people who moved were less likely to have adverse birth outcomes than those who did not [50]. Most pregnant women have no movement during pregnancy, and a few pregnant women have short-distance movement during pregnancy. Using the registration address at birth, there may be registration errors. However, there is no significant difference in the exposure concentration of air pollutants during pregnancy, so the address registered at the birth of the fetus can be a good substitute for the residential address during pregnancy [51].

Our study has several advantages. First, we conducted a systematic review and meta-analysis. Compared with individual studies, the evidence level of systematic reviews and meta-analysis is relatively high. Second, we divided OFCs into CL, CP, CL/P and analyzed them with air pollutants respectively. Finally, among the 11 articles we included, 10 studies were low risk of bias and only 1 study was high risk of bias. After adjusting for them, we found that the heterogeneity was lower than before.

There are some limitations in our research. First of all, we study the high heterogeneity between some literatures, which is related to the limited number of literatures, different geographical locations, different exposure methods, and large research time span. Secondly, there may be differences in the diagnosis of diseases in different studies. There are no trained nurses to inquire about prenatal exposure factors, and professional doctors use relevant scales to classify and evaluate diseases [52, 53]. Third, in the included literature, there is not enough information for dose response assessment, and we have not been able to assess whether there is a linear relationship between pollutant exposure concentration and the occurrence of OFCs. Fourthly, different studies have different exposure assessment methods. Marshall EG et al., Hansen CA et al. and Liu FH et al. used air quality monitoring stations to monitor the exposure assigned by individuals within a specified radius around, which may lead to measurement errors in exposure concentration, which may cause errors in risk assessment and result deviations [25, 26, 30]. Finally, we used the pregnancy address as the exposure point all studies in this meta-analysis, without considering the possibility of mobility during pregnancy.

## Conclusion

In conclusion, the results of this study show that there is a positive correlation between PM_2.5_, PM_10_, O_3_ and OFCs, and there is no statistical significance between residual air pollutants and OFCs. However, this association may be affected by the study area and research-related exposure methods, which are important factors causing heterogeneity, and further large-scale cohort studies are needed to verify this association. It is recommended that future public health work should minimize exposure of pregnant women to related air pollutants.

### Electronic supplementary material

Below is the link to the electronic supplementary material.

Supplementary Material 1

## Data Availability

Any data in this study can be obtained from the corresponding author upon reasonable request. The final manuscript was read and approved by all authors.
