# Supplementary material for: Association between gestational exposure and risk of orofacial clefts: a systematic review and meta-analysis
Source: BMC Pregnancy Childbirth. 2023 Dec 1;23:829. doi: 10.1186/s12884-023-06104-4 (PMC10691060; doi:10.1186/s12884-023-06104-4)
Supplement: Supplementary file 1 — Supplementary Material 1 [file 12884_2023_6104_MOESM1_ESM.docx]

**supplementary materials**

1.search formulation

1.1pub med

(("cleft lip"[MeSH Terms] OR ("cleft"[All Fields] AND "lip"[All Fields]) OR "cleft lip"[All Fields]) OR ("cleft lip"[MeSH Terms] OR ("cleft"[All Fields] AND "lip"[All Fields]) OR "cleft lip"[All Fields] OR "harelip"[All Fields]) OR ("cleft palate"[MeSH Terms] OR ("cleft"[All Fields] AND "palate"[All Fields]) OR "cleft palate"[All Fields] OR ("cleft"[All Fields] AND "palates"[All Fields]) OR "cleft palates"[All Fields]) OR ("cleft palate"[MeSH Terms] OR ("cleft"[All Fields] AND "palate"[All Fields]) OR "cleft palate"[All Fields] OR "palatoschisis"[All Fields]) OR (orofacial[All Fields] AND clefts[All Fields])) AND (("air pollution"[MeSH Terms] OR ("air"[All Fields] AND "pollution"[All Fields]) OR "air pollution"[All Fields] OR ("air"[All Fields] AND "pollutions"[All Fields]) OR "air pollutions"[All Fields]) OR ("particulate matter"[MeSH Terms] OR ("particulate"[All Fields] AND "matter"[All Fields]) OR "particulate matter"[All Fields]) OR ("sulphur dioxide"[All Fields] OR "sulfur dioxide"[MeSH Terms] OR ("sulfur"[All Fields] AND "dioxide"[All Fields]) OR "sulfur dioxide"[All Fields]) OR ("nitrogen dioxide"[MeSH Terms] OR ("nitrogen"[All Fields] AND "dioxide"[All Fields]) OR "nitrogen dioxide"[All Fields]) OR ("carbon monoxide"[MeSH Terms] OR ("carbon"[All Fields] AND "monoxide"[All Fields]) OR "carbon monoxide"[All Fields]) OR ("ozone"[MeSH Terms] OR "ozone"[All Fields]))

1.2 Embase

1.((Cleft Lip or Harelip or Cleft Palate or palatoschisis or orofacial cleft or (cleft lip with or without cleft palate)

2.(Air Pollution or Air Quality or Sulfur Dioxide or Nitrogen Dioxide or Carbon Monoxide or Particulate Matter or ozone)

1 and 2

1.3 web of science

1.Air Pollution (Topic) or Air Quality (Topic) or Sulfur Dioxide (Topic) or Nitrogen Dioxide (Topic) or Carbon Monoxide (Topic) or Particulate Matter (Topic) or ozone (Topic)

2.Cleft Lip (Topic) or Harelip (Topic) or Cleft Palate (Topic) or palatoschisis (Topic) or orofacial cleft (Topic) or cleft lip with or without cleft palate (Topic)

3.#2 AND #1

**Association between PM10 and CL**


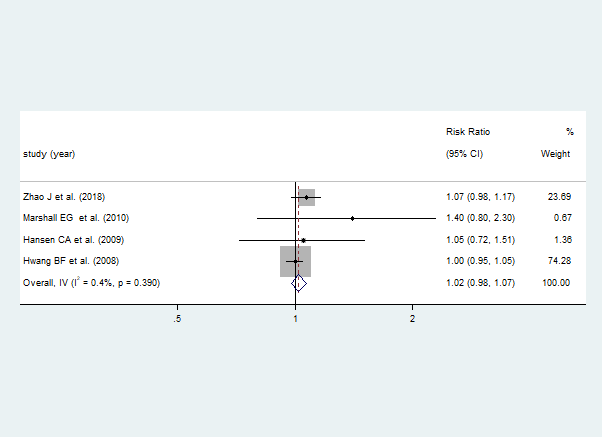


**Association between PM10 and CP**


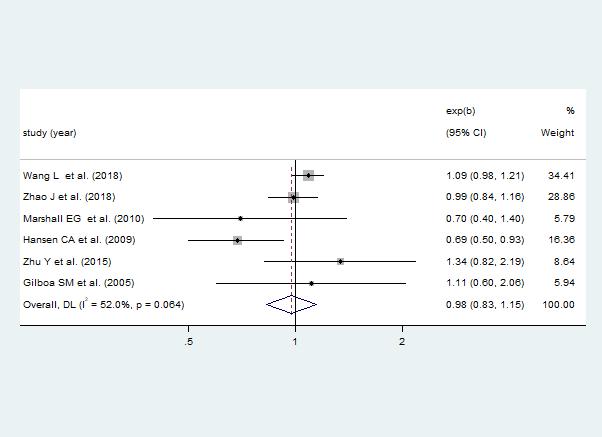


**Association between PM10 and CL/P**


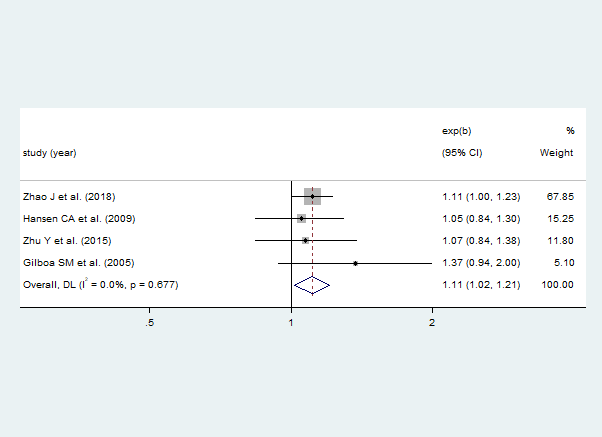


**Association between PM2.5 and CL**


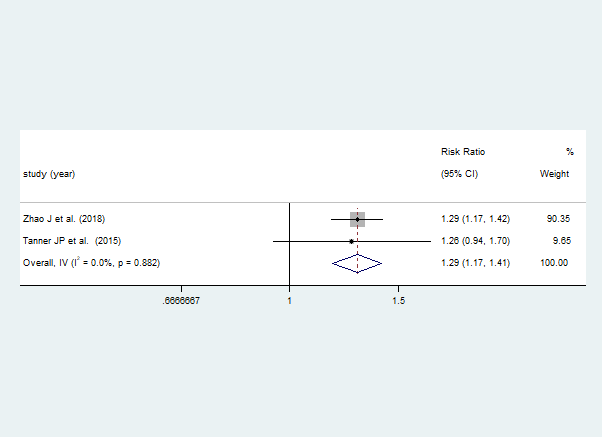


**Association between PM2.5 and CP**


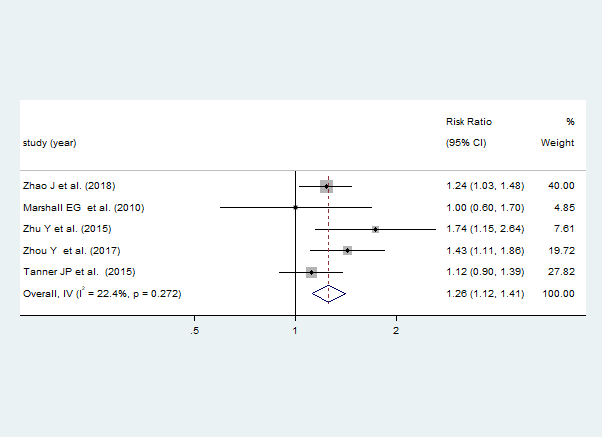


**Association between PM2.5 and CP（sensitivity analysis）**


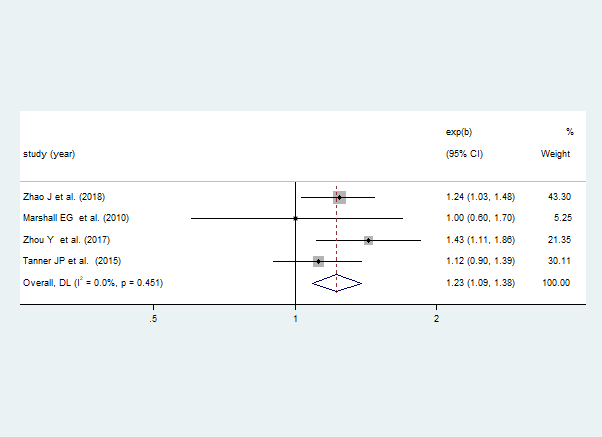


**Association between PM2.5 and CL/P**


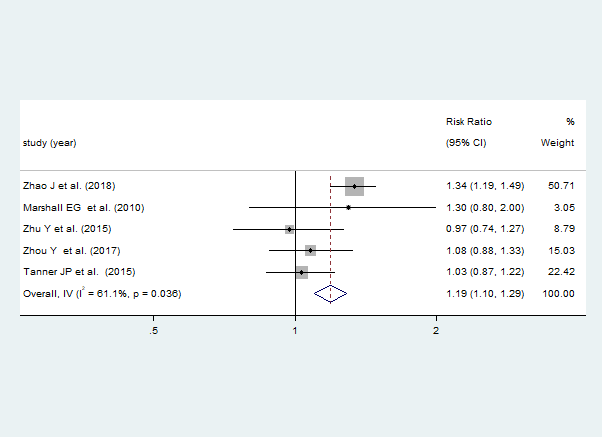


**Association between SO2 and CL**


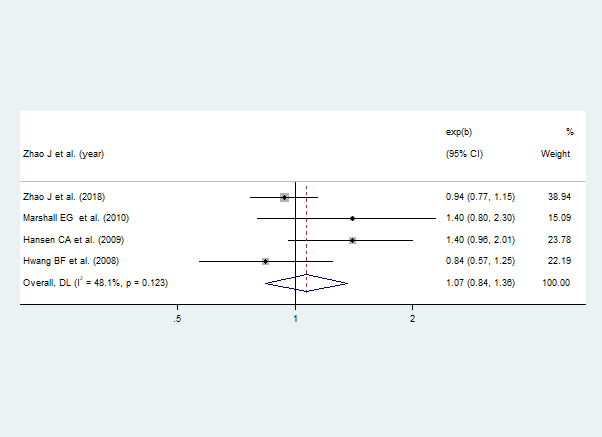


**Association between SO2 and CP**


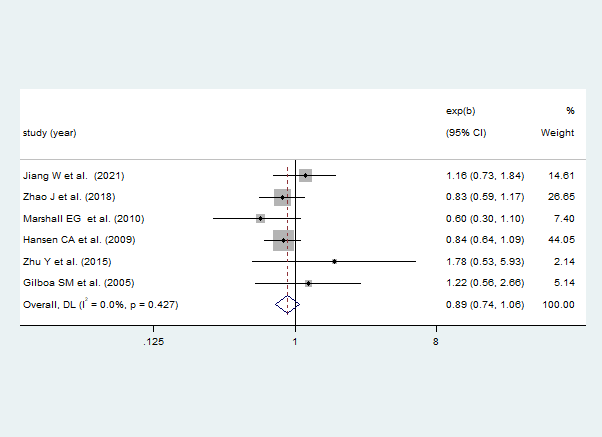


**Association between SO2 and CL/P**


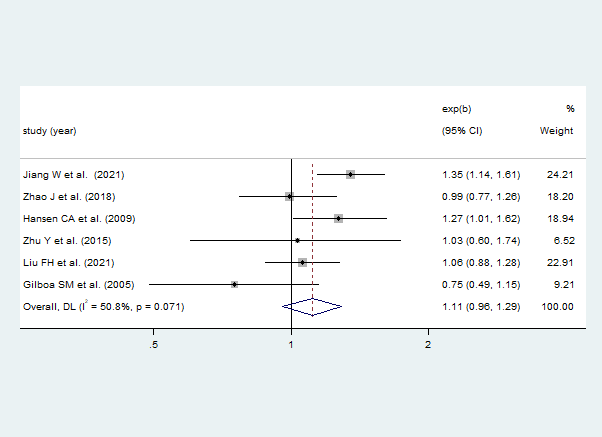


**Association between O3 and CL**


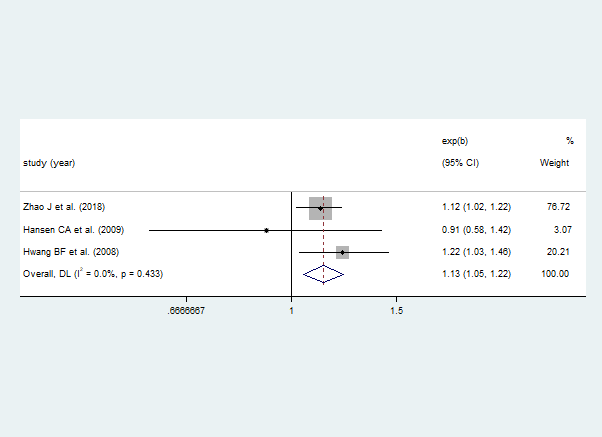


**Association between O3 and CP**


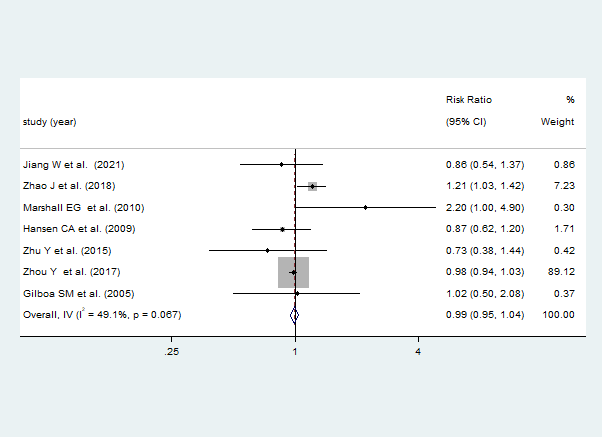


**Association between O3 and CL/P**


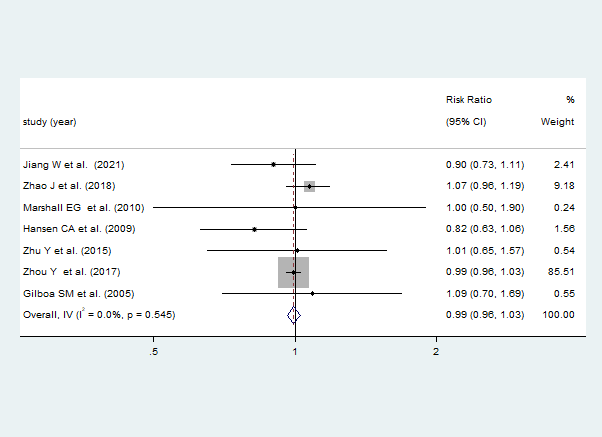


**Association between CO and CL**


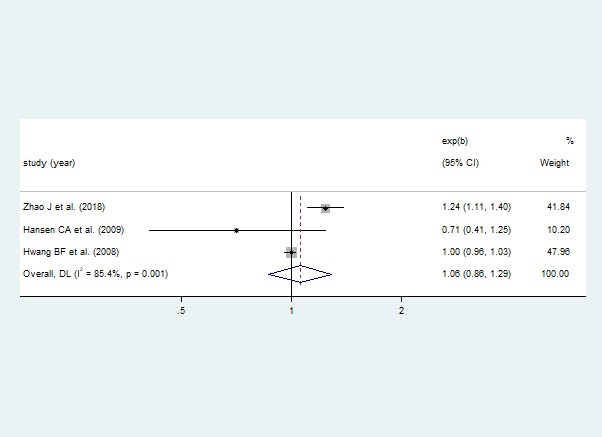


**Association between CO and CP**


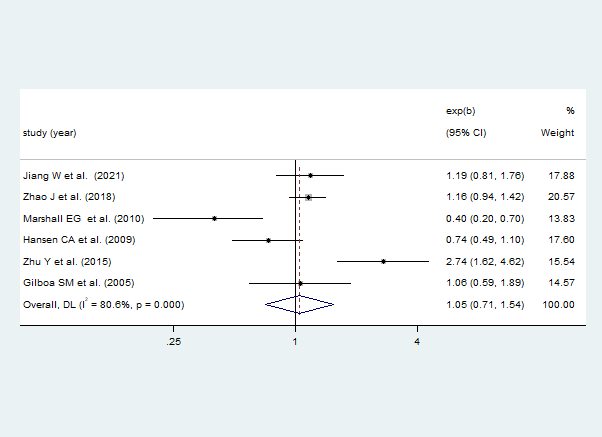


**Association between CO and CL/P**


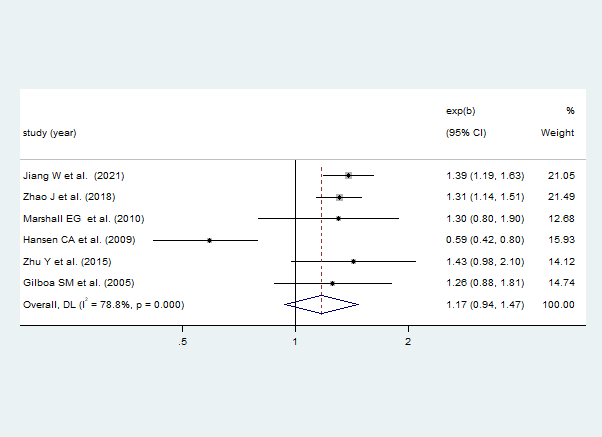


**Association between NO2 and CL**


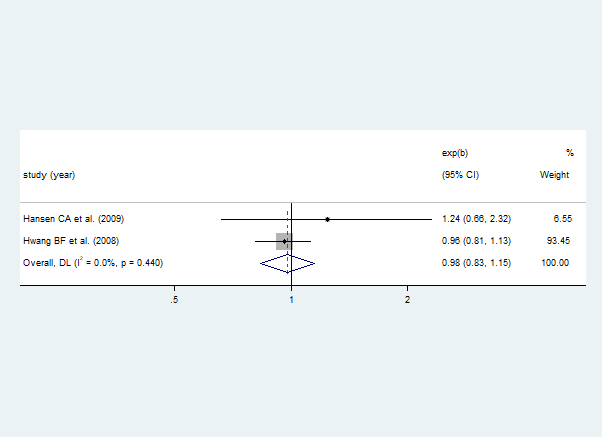


**Association between NO2 and CP**


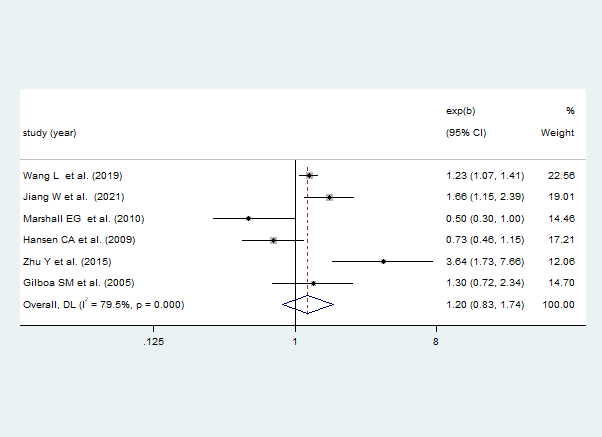


**Association between NO2 and CL/P**


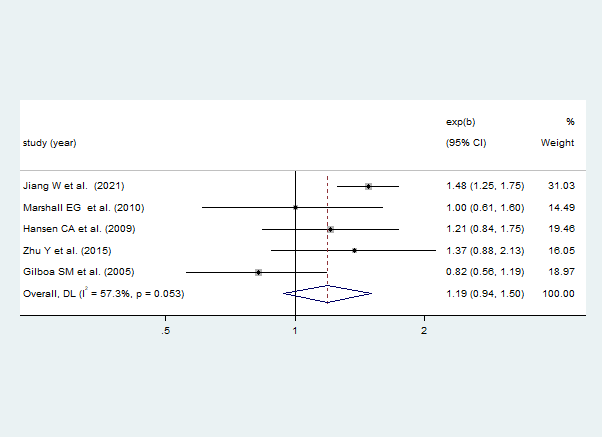


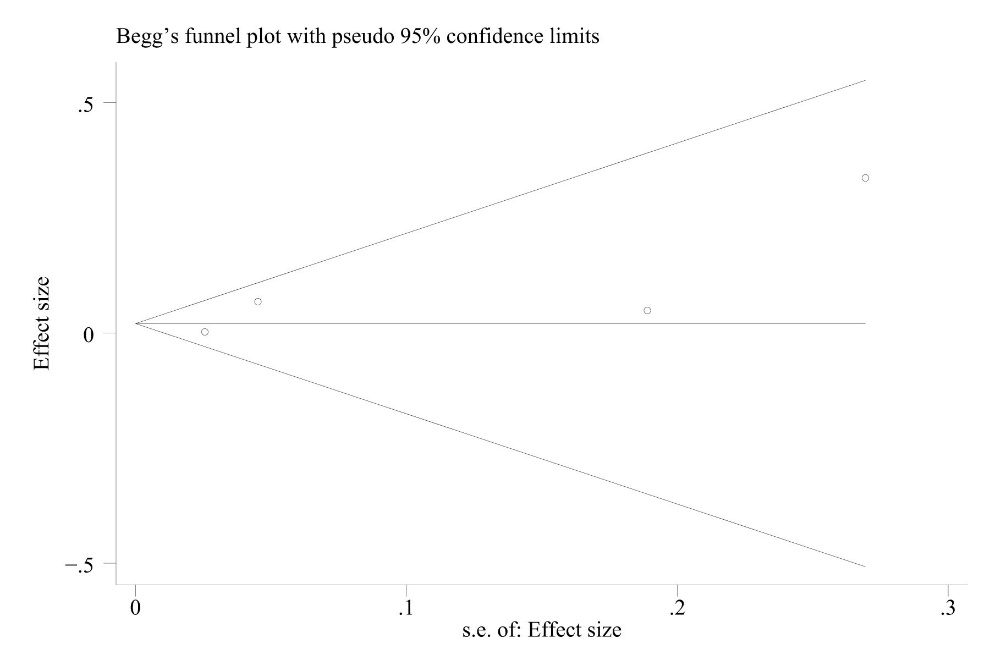


**Publication bias of PM10 and CL**


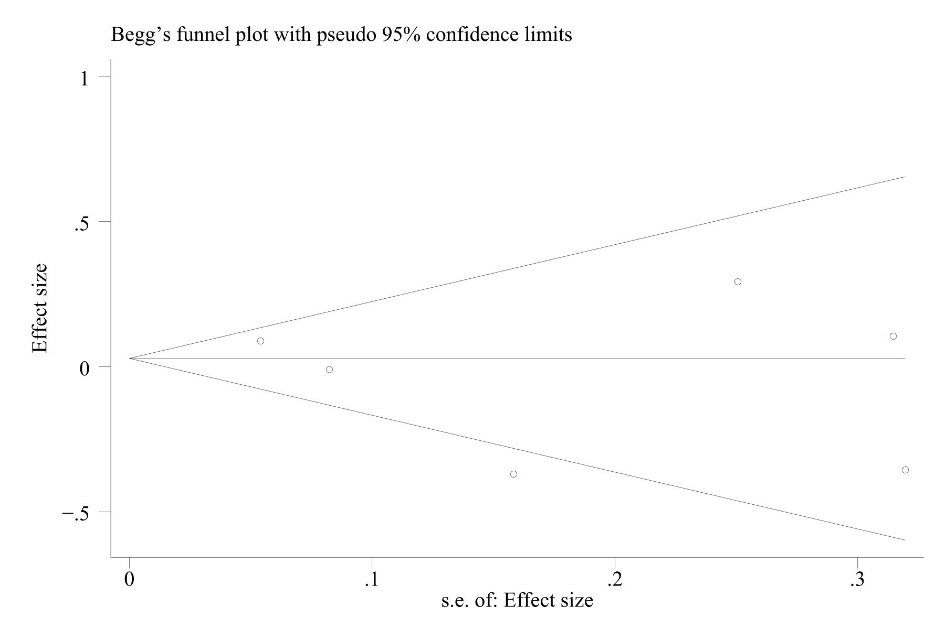


**Publication bias of PM10 and CP**


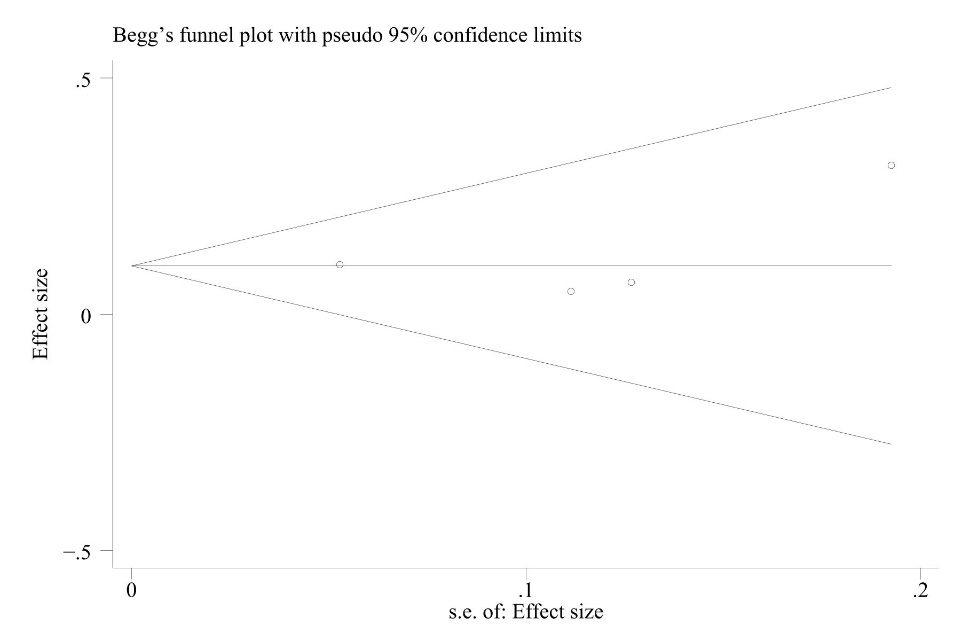


**Publication bias of PM10 and CL/P**


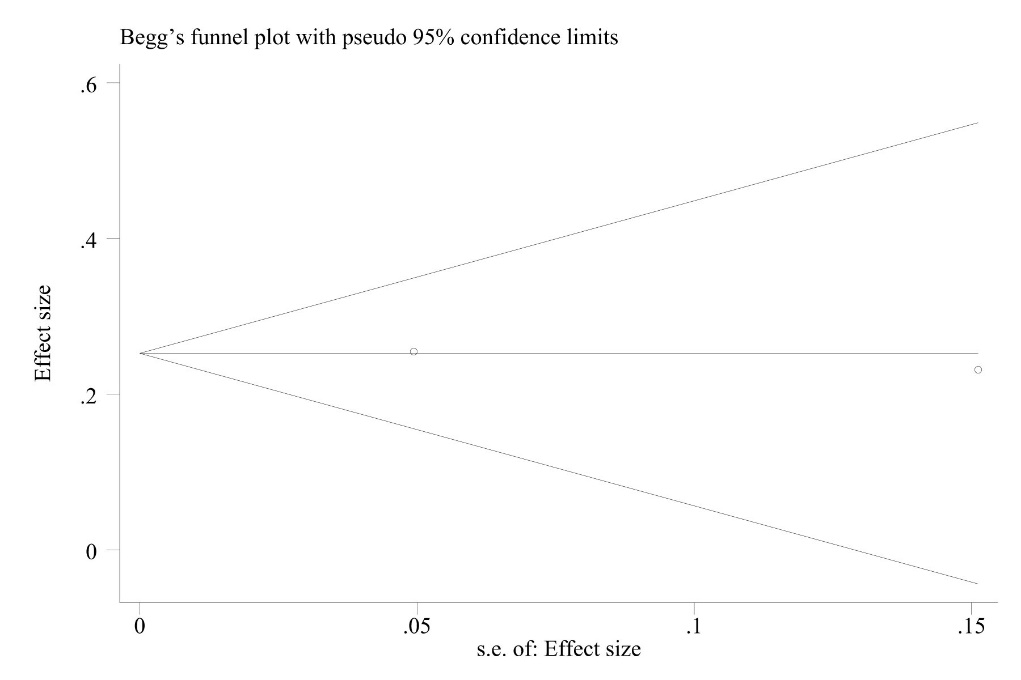


**Publication bias of PM2.5 and CL**


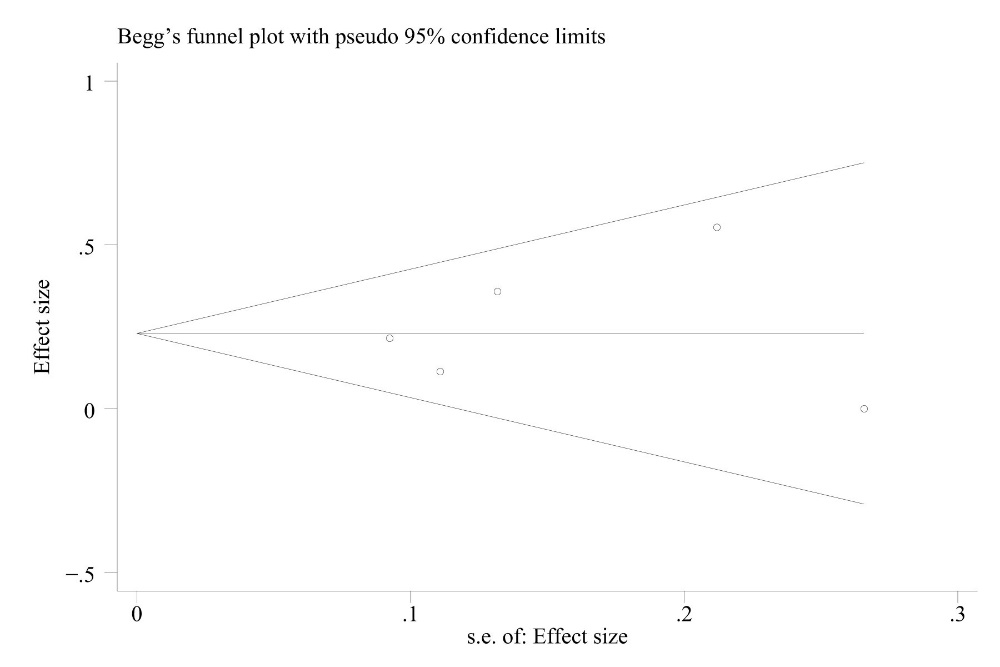


**Publication bias of PM2.5 and CP**


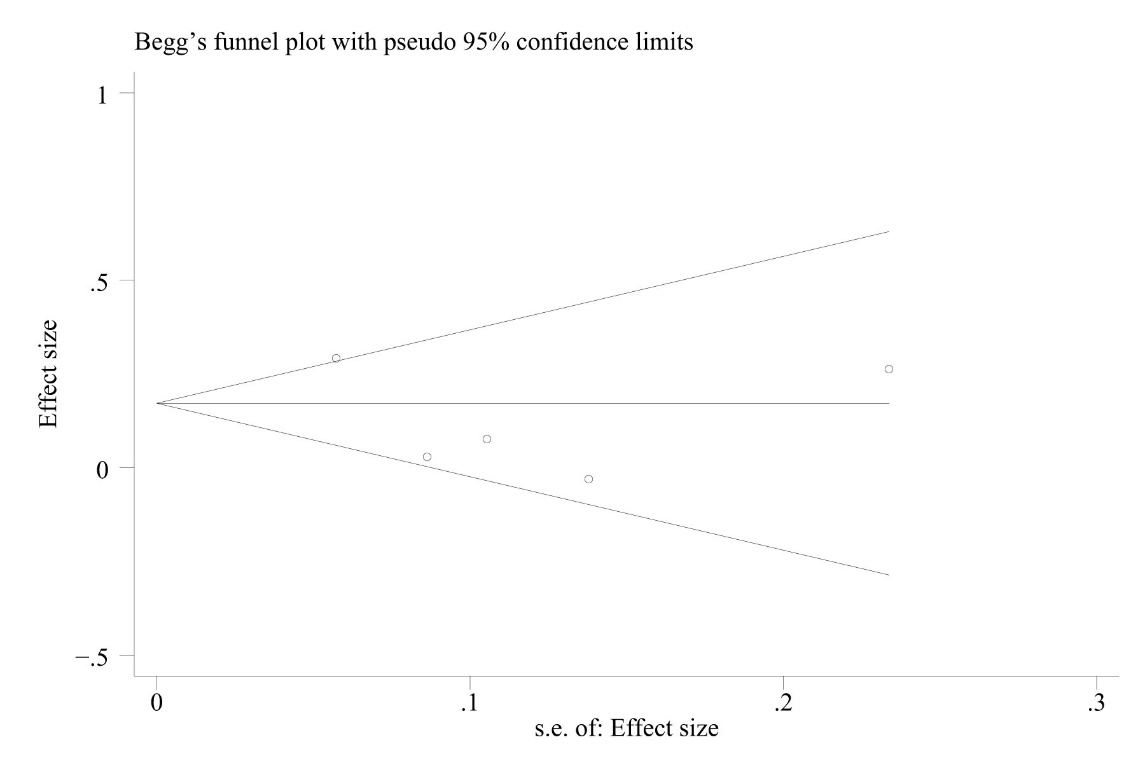


**Publication bias of PM2.5 and CL/P**


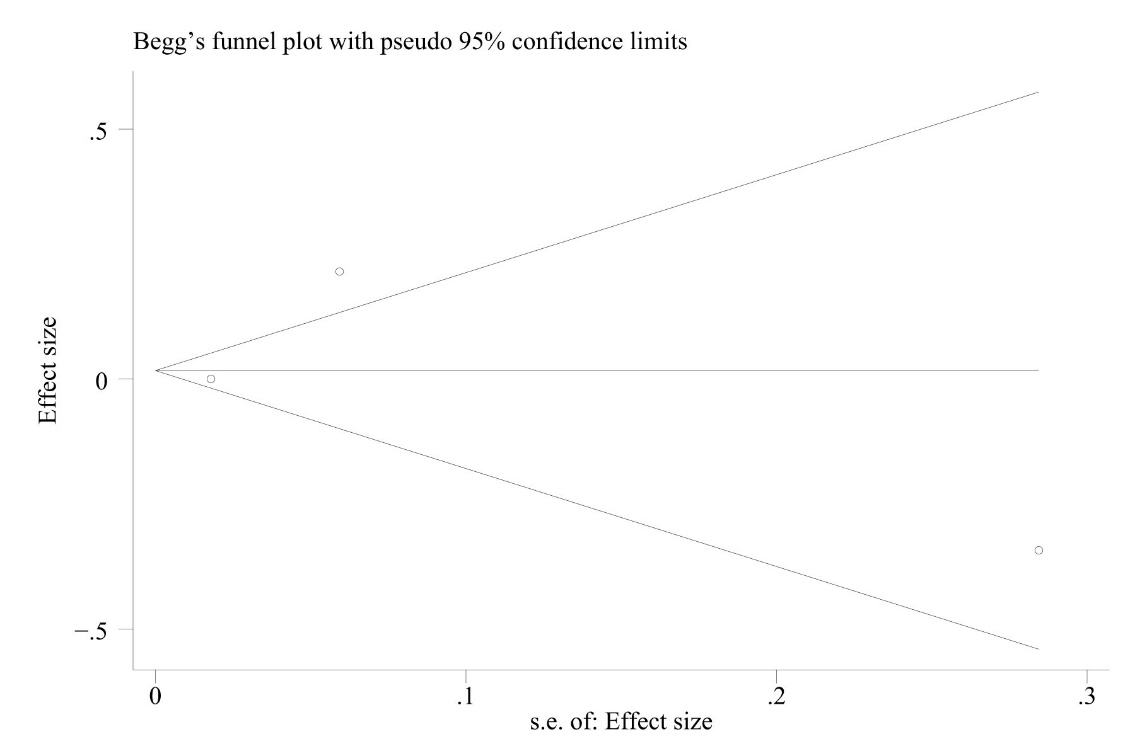


**Publication bias of CO and CL**


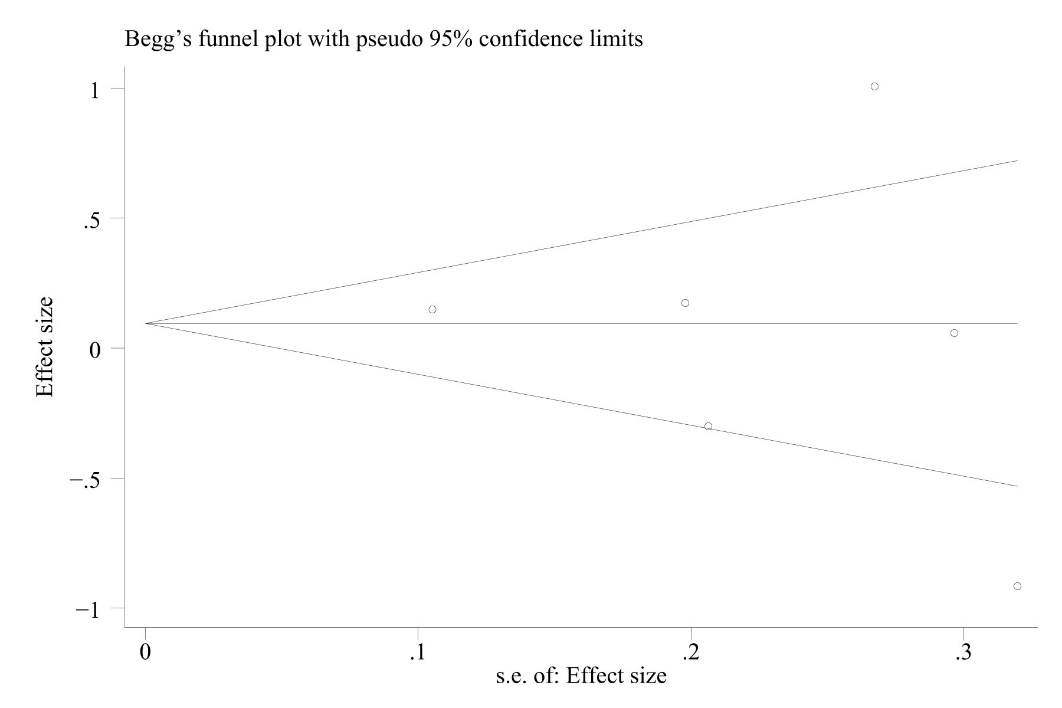


**Publication bias of CO and CP**


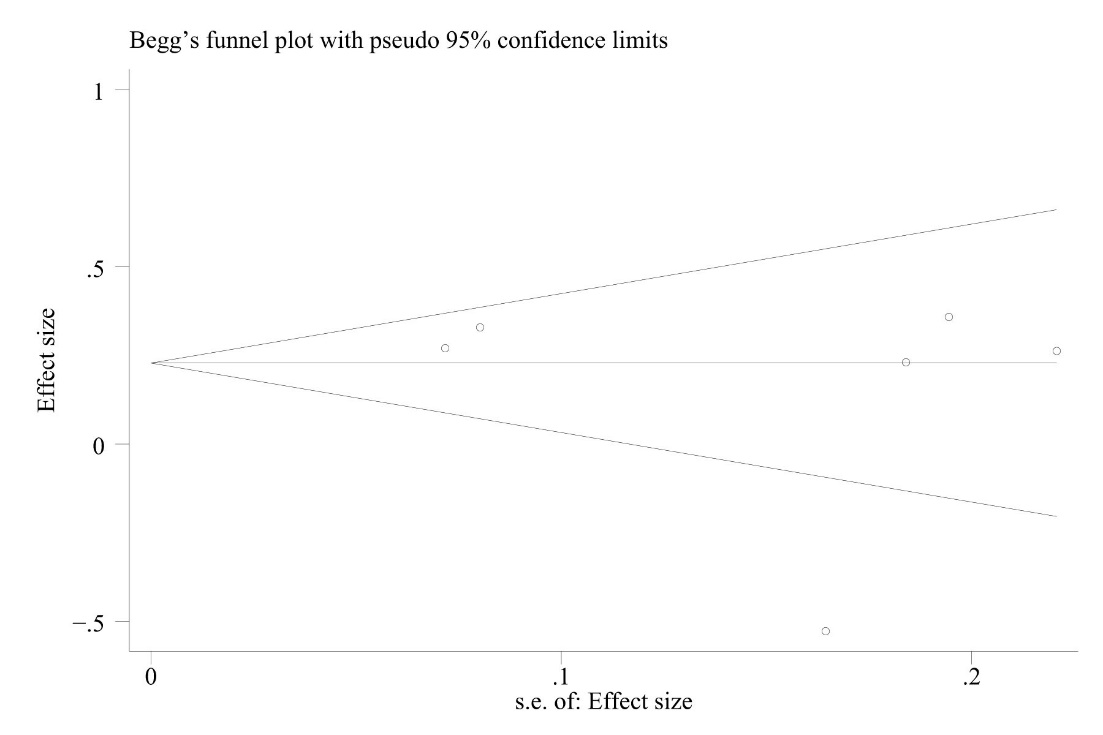


**Publication bias of CO and CL/P**


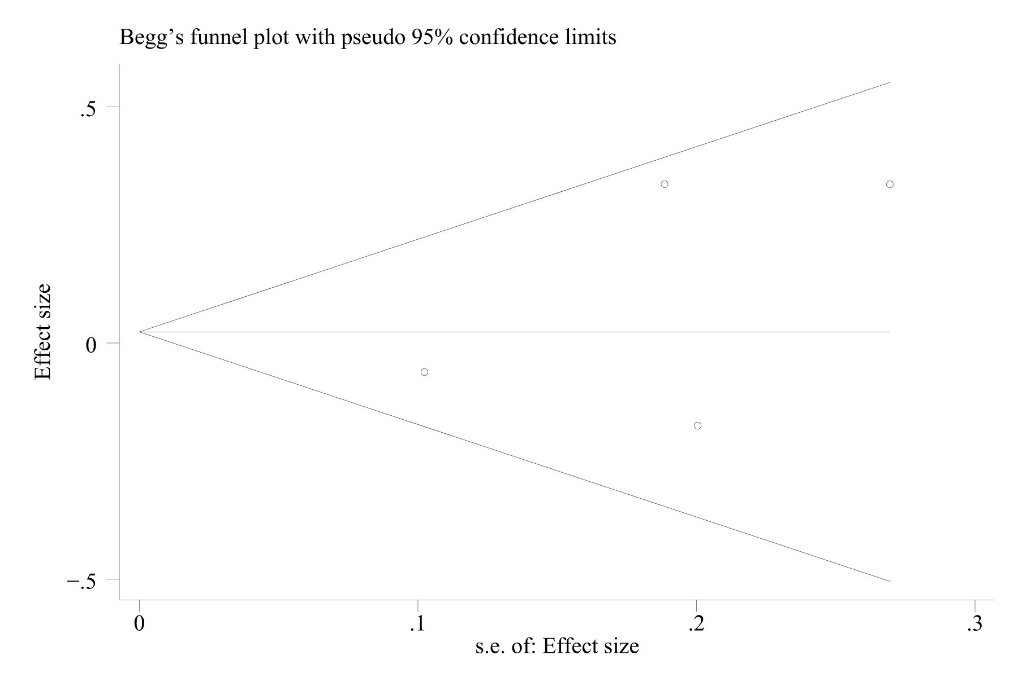


**Publication bias of SO2 and CL**


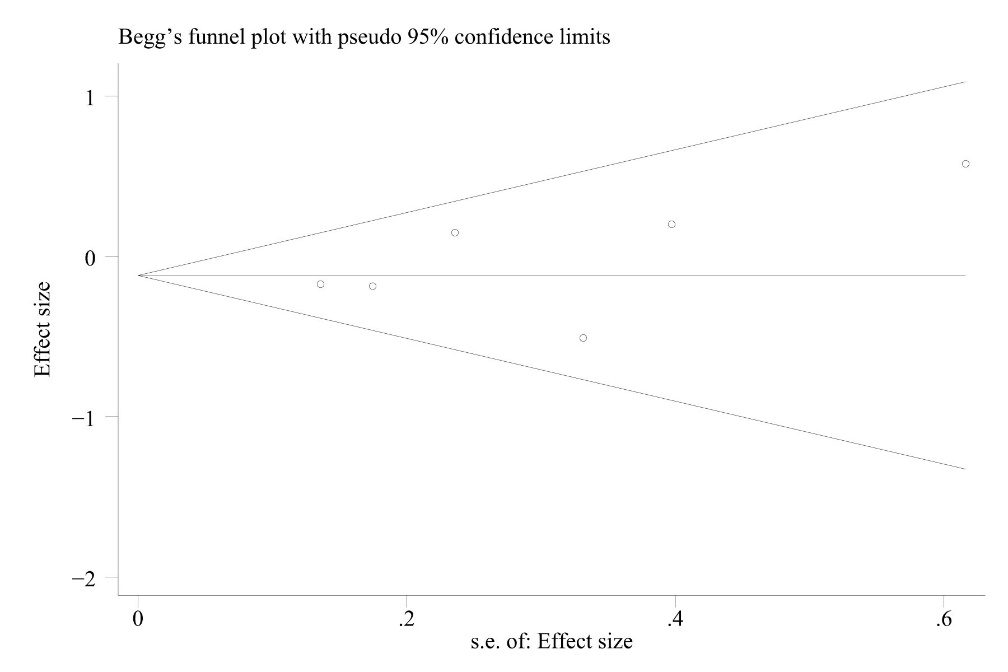


**Publication bias of SO2 and CP**


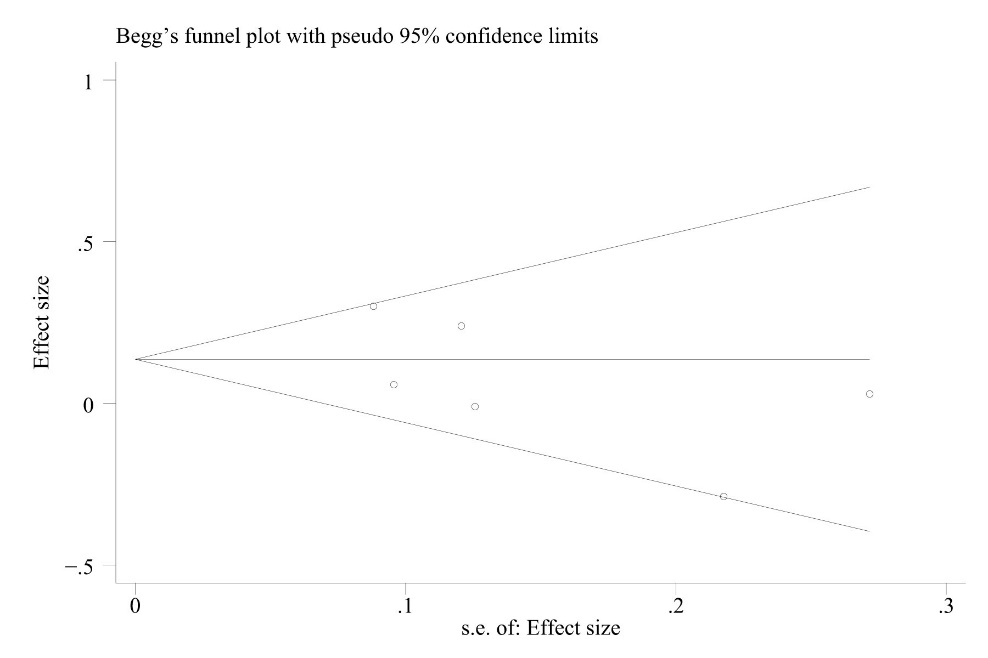


**Publication bias of SO2 and CL/P**


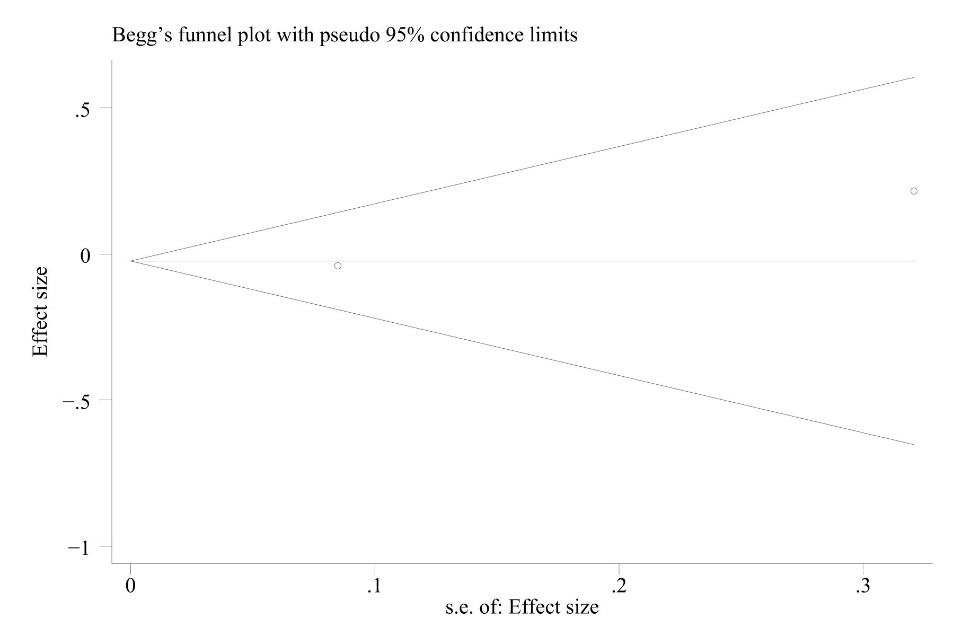


**Publication bias of NO2 and CL**


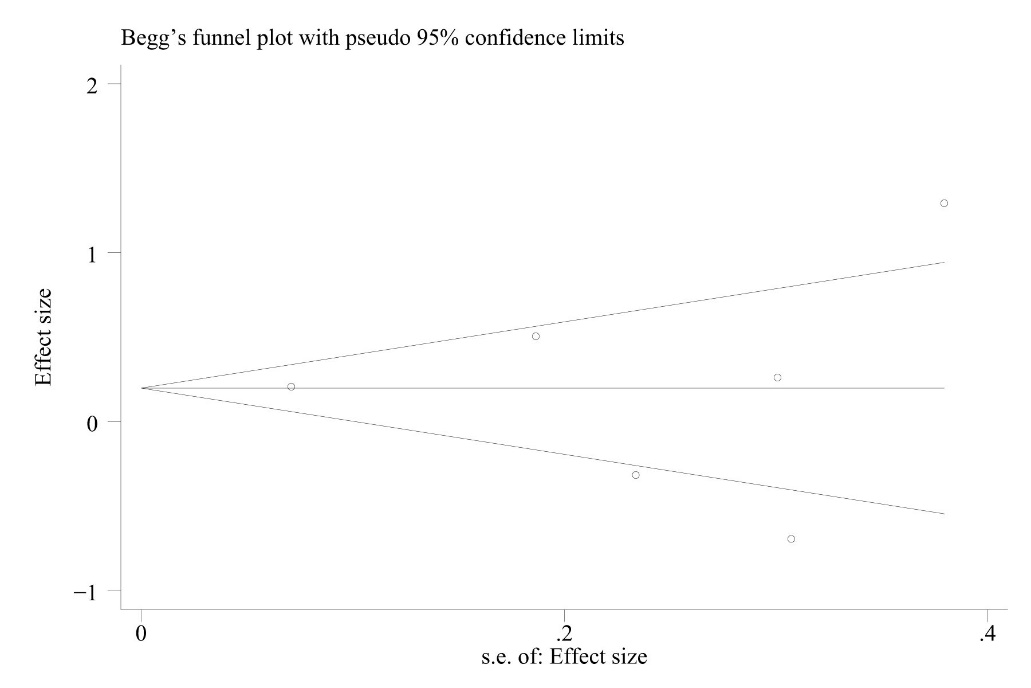


**Publication bias of NO2 and CP**


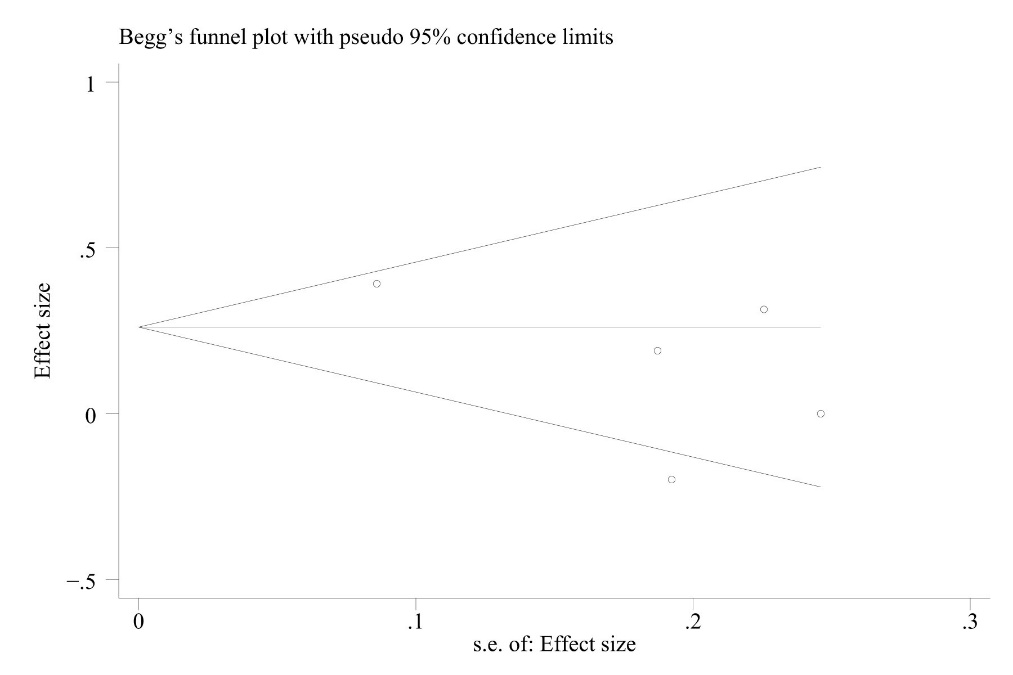


**Publication bias of NO2and CL/P**
